# Giant Ganglioneuroma of the Lumbar Spine: A Rare Cause of Radiculopathy

**DOI:** 10.1155/2024/9477892

**Published:** 2024-05-23

**Authors:** Lina Altalhi, Abdulaziz Alayyaf, Mohammed Bin-Mahfooz, Duaa Alhumoudi, Ali Alkhaibary, Fahd AlSufiani, Ali H. Alassiri, Saad AlQahatani, Sami Khairy, Ahmed Alkhani

**Affiliations:** ^1^ College of Medicine and Medical Science Arabian Gulf University, Manama, Bahrain; ^2^ College of Medicine Prince Sattam bin Abdulaziz University, Riyadh, Saudi Arabia; ^3^ College of Medicine King Saud Bin Abdulaziz University for Health Sciences, Riyadh, Saudi Arabia; ^4^ College of Medicine King Saud University, Riyadh, Saudi Arabia; ^5^ King Abdullah International Medical Research Center, Riyadh, Saudi Arabia; ^6^ Division of Neurosurgery Department of Surgery King Abdulaziz Medical City Ministry of National Guard-Health Affairs, Riyadh, Saudi Arabia; ^7^ Department of Pathology and Laboratory Medicine King Abdulaziz Medical City Ministry of National Guard-Health Affairs, Riyadh, Saudi Arabia

## Abstract

**Background:**

Ganglioneuroma (GN) is a rare, benign tumor that originates from neural crest cells and can potentially affect any anatomical site within the sympathetic nervous system. Typically, GNs are more frequently reported in children and young adults, with a slightly higher prevalence in females. We are reporting a rare case of a giant lumbar spine ganglioneuroma by outlining the clinical presentation, radiological finding, management, and outcome. *Case Description*. A 37-year-old female presented with low back pain radiating to the right lower limb for few years. Neurological examination revealed bilateral lower limb hyperreflexia (+3). Lumbar spine CT and MRI revealed a right paravertebral soft tissue lesion with heterogeneous signal intensity and enhancement at L1 to L3. The patient underwent complete resection of the lesion via a retroperitoneal approach. The surgery was uneventful. The histopathological sections were suggestive of mature ganglioneuroma. She was discharged in stable condition with follow-up at neurosurgery clinic.

**Conclusion:**

Giant ganglioneuromas are rare, benign tumors of sympathetic neurons. Complete surgical resection is the most effective therapeutic option for ganglioneuroma to avoid recurrence. Given the benign nature of ganglioneuroma, chemotherapy and radiotherapy tend to have a limited role following surgical resection.

## 1. Introduction

Ganglioneuroma is a rare and well-differentiated neoplasm comprising ganglion cells and Schwann cells, which arises from neural crest cells and exhibits a slow-growing behavior. It can potentially affect any anatomical site within the sympathetic nervous system, ranging from the skull base to the pelvis. Peripheral neuroblastic tumors range from benign to malignant. The clinical manifestations and symptoms of spinal ganglioneuromas differ according to their specific location along the spine [1].

The International Neuroblastoma Pathology Classification is based on the morphological features of neuroblastic tumors (NTs) (i.e., neuroblastoma, ganglioneuroblastoma, and ganglioneuroma) [2]. Histopathologically, ganglioneuromas show admixture of ganglion cell and Schwann cells that vary in distribution and number [2]. It may also harbor granular pigments of neuromelanin [2]. Typically, ganglioneuromas are detected more frequently in young adults, with a slightly higher prevalence in females. The cervical spine is the most frequent location for spinal ganglioneuromas, followed by the thoracic spine and then the lumbar spine [3]. We are reporting a rare case of a giant lumbar spine ganglioneuroma by outlining the clinical presentation, radiological finding, management, and outcome.

## 2. Case Description

### 2.1. Clinical Presentation

A 37-year-old female presented with low back pain radiating to the right lower limb for 4 years. The pain is mostly continuous and exacerbated by movements (walking). The patient denied urinary/stool incontinence, saddle anesthesia, or numbness.

Physical examination revealed hyperreflexia in the lower limbs (+3). The muscle power and sensation were intact. Straight leg raising and gait were unremarkable.

### 2.2. Neuroradiological Imaging

**Figure 1:**
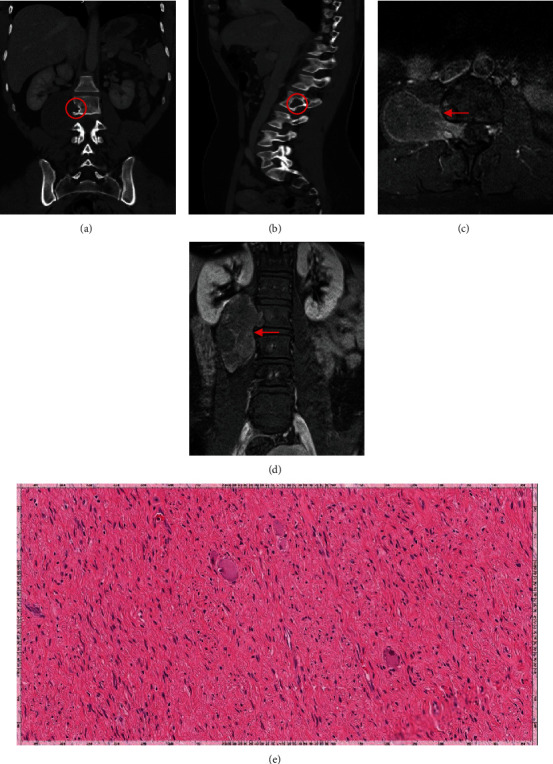
(a, b) Coronal and sagittal lumbar CT (bone window) demonstrating an eccentric, expansile lytic lesion at the posterior aspect of the right L2 vertebral body extending along the right psoas muscle. It is causing mass effect on the right neural foramen. (c, d) Axial and coronal lumbar MRI with contrast showing a right paravertebral soft tissue lesion with heterogeneous signal intensity and enhancement at L1 to L3. There is spinal canal component at L2-L3 right neural foramen and epidural space involving the exiting nerve. It invaginates into the right psoas muscle and causes scalloping of the right vertebral body of L2 and L3. It measures about 9 × 6 × 4 cm in maximum diameters. (e) Hematoxylin and eosin stain (high power; magnification ×200). Ganglioneuroma, mature: admixture of few mature ganglion cells and abundant atypical Schwann cells.

Lumbar spine CT and MRI revealed an eccentric expansile lytic soft tissue lesion at the posterior aspect of the right L2 vertebral body extending along the right psoas muscle with mass effect on the right neural foramen (Figures 1(a), 1(b), 1(c), and 1(d)).

### 2.3. Surgical Approach

Given the radiological findings, the patient underwent resection of the lesion via a retroperitoneal approach. The patient was placed in a lateral decubitus position with the right side up. A right flank linear incision was done, and the fascia and abdominal muscle were dissected. After that, retroperitoneal dissection was performed until the tumor in the psoas muscle was felt. The psoas muscle was opened in a linear incision and the tumor was exposed; it was entirely dissected from its capsule. All dissection planes were extraperitoneal. Neurophysiological monitoring during and after the surgery was baseline and showed no abnormality or interruptions. The patient tolerated the surgery well.

### 2.4. Histopathological Findings

Grossly, the tumor was yellowish with a tan texture. It was soft with no areas of calcification. The histopathological sections of the tumor were suggestive of mature ganglioneuroma. It showed admixture of few mature ganglion cells and abundant bland Schwann cells (Figure 1(e)).

### 2.5. Outcome and Follow-Up

Upon discharge, the patient was doing well with no postoperative complications. She reported decreased sensation at L2 distribution. Her symptoms improved gradually. She was discharged in stable condition with follow-up at neurosurgery clinic. 12 months later, the patient reported significant improvement of her symptoms.

## 3. Discussion

Of all central nervous system tumors, the incidence of ganglioneuromas is estimated to be less than 0.5% [4, 5]. Prior studies have demonstrated that the most frequent location of GNs is the posterior mediastinum and the retroperitoneal space representing 41.5% and 37.5%, respectively [6–8]. Furthermore, spinal GNs constitute less than 10% of all ganglioneuromas cases, with the cervical spine being the most frequently affected region followed by even rarer occurrences in the thoracic and lumbar spine [3, 9].

While lumbar spine ganglioneuromas can be incidentally discovered, others may present with motor/sensory deficits, abnormal gait, bowel/urinary disturbances, and lower back pain [3, 10–14]. In addition, secondary scoliosis was reported in three cases [10–12]. In the present case, the patient presented with radicular low back pain that persisted for few years and hyperreflexia.

Neuroradiological imaging is pivotal in the initial assessment of such tumors as they can provide valuable information on the extent of the tumor, regional infiltration, source of origin, and the presence of calcification [15]. On magnetic resonance imaging, ganglioneuromas often display isointensity or hypointensity on T1-weighted images compared to hyperintensity on T2-weighted images. In contrast-enhanced T1-weighted images, it can exhibit some heterogeneity. Additionally, contrast-enhanced CT often displays homogeneity and a relatively well-encapsulated mass [16].

Complete surgical resection is the most effective therapeutic option for ganglioneuroma to avoid recurrence [17]. Different surgical approaches were described in the literature for spinal tumors, including dual approach in which there is an anterior or retroperitoneal stage and a posterior laminectomy in attempt of total resection. However, dual technique is unfavorable as it is associated with high morbidity [18]. Alternatively, the patient in the present case underwent a lateral retroperitoneal transpsoas approach (single-position surgery). Retroperitoneal approaches enable simultaneous access to both anterior and posterior columns of the spine without having to intraoperatively reposition the patient or stage the surgery, and it is associated with better outcomes [19].

Given the benign nature of this tumor, chemotherapy and radiotherapy have a limited role following surgical resection [20]. Generally, if complete tumor resection is carried out, the prognosis of ganglioneuroma is highly favorable regardless of its location [20]. In the present case, the management comprised en bloc resection of the tumor which resulted in excellent outcome.

Few cases were reported on spinal ganglioneuroma in the literature. Jeong et al. described a patient with L5 nerve root ganglioneuroma [1]. Due to the inoperable location of the tumor, medial facetectomy followed by a biopsy was performed [1]. Takebayashi et al. reported the first case of lumbar ganglioneuroma found in continuity between the intradural and extradural spaces [11]. López et al. reported a case of intradural ganglioneuroma masquerading as lumbar disc herniation [12]. The tumor was discovered intraoperatively [12]. It was sent to histopathology and the results were consistent with ganglioneuroma [12].

## 4. Conclusion

Giant ganglioneuromas are rare, benign tumors of sympathetic neurons. The majority of cases occur within the thoracoabdominal region. The highest prevalence is observed among children and adolescents. When feasible, total resection is the most effective approach to manage ganglioneuromas and avoid recurrence. A lateral retroperitoneal approach can be used to resect such complex lesions and allows access to both anterior and posterior columns of the spine. Herein, we presented a rare case of lumbar radiculopathy secondary to a giant ganglioneuroma resected via a retroperitoneal approach.

## Data Availability

The data are available upon request.
